# Cerebral Blood Flow and Its Connectivity Deficits in Mild Traumatic Brain Injury at the Acute Stage

**DOI:** 10.1155/2020/2174371

**Published:** 2020-07-01

**Authors:** Fengfang Li, Liyan Lu, Song'an Shang, Huiyou Chen, Peng Wang, Nasir Ahmad Haidari, Yu-Chen Chen, Xindao Yin

**Affiliations:** Department of Radiology, Nanjing First Hospital, Nanjing Medical University, Nanjing, China

## Abstract

**Objective:**

The influence of cognitive impairment after mild traumatic brain injury (mTBI) on cerebral vascular perfusion has been widely concerned, yet the resting-state cerebral blood flow (CBF) connectivity alterations based on arterial spin labeling (ASL) in mild traumatic brain injury (mTBI) remain unclear. This study investigated region CBF and CBF connectivity features in acute mTBI patients, as well as the associations between CBF changes and cognitive impairment.

**Materials and Methods:**

Forty-five acute mTBI patients and 42 health controls underwent pseudocontinuous arterial spin labeling (pCASL) perfusion magnetic resonance imaging (MRI). The alterations in regional CBF and relationship between the CBF changes and cognitive impairment were detected. The ASL-CBF connectivity of the brain regions with regional CBF significant differences was also compared between two groups. Neuropsychological tests covered seven cognitive domains. Associations between the CBF changes and cognitive impairment were further investigated.

**Results:**

Compared with the healthy controls, the acute mTBI patients exhibited increased CBF in the bilateral inferior temporal gyrus (ITG) and decreased CBF in the right middle frontal gyrus (MFG), the bilateral superior frontal gyrus (SFG), and the right cerebellum posterior lobe (CPL). In the mTBI patients, significant correlations were identified between the CBF changes and cognitive impairment. Importantly, the acute mTBI patients exhibited CBF disconnections between the right CPL and right fusiform gyrus (FG) as well as bilateral ITG, between the left SFG and left middle occipital gyrus (MOG), and between the right SFG and right FG as well as right parahippocampal gyrus.

**Conclusion:**

Our results suggest that acute mTBI patients exhibit both regional CBF abnormalities and CBF connectivity deficits, which may underlie the cognitive impairment of the acute mTBI patients.

## 1. Introduction

Mild traumatic brain injury (mTBI), also commonly referred to as concussion, is recognized as a major public health concern worldwide [1]. A large number of mTBI patients may have measurable deficits in some cognitive areas, mainly including visuospatial and executive functioning, attention, language, and memory [2, 3], which can interfere with daily life. It is estimated that up to 40-50% of mTBI patients still have cognitive deficit symptoms 3 months after injury and 10-15% at even years after injury [4]. However, the specific mechanism that leads to brain deficits and ultimately cognitive impairment in mTBI patients is still controversial and remains to be elucidated. In addition to structural and functional brain changes, imaging studies using single-photon emission computed tomography (SPECT) and positron emission tomography (PET) have shown abnormal cerebral blood flow (CBF) and cerebral glucose metabolism in multiple brain regions of mTBI patients at different stages [5, 6]. Furthermore, there are also indications that alterations of the CBF are associated with persistent symptoms and impaired cognitive functioning [7]. Meanwhile, several resting-state CBF alterations have been associated with the core clinical cognitive impairments symptoms of mTBI [8, 9]. Although SPECT, PET, and arterial spin labeling (ASL) can all evaluate brain hemodynamics, SPECT and PET are nuclear medicine techniques that require using invasive radioactive tracers, which limit repeated testing. On the contrary, ASL provides a noninvasive means for estimating CBF, which has been shown to be a robust method of assessing microvascular perfusion, and is less influenced by pathologic damage to the blood-brain barrier, including TBI [10].

Profiting from a short acquisition time and endogenous contrast agent, ASL perfusion sequence is used to evaluate CBF at resting-state and could serve as a marker of functional activation albeit, which achieves a direct measure of regional CBF and independent of complicated calculations [11, 12]. Using this technique, the alterations of CBF in TBI have been explored in animal models [12] and human studies [13], but few studies focused on mTBI, especially the acute mTBI. Recently, chronic TBI [13] and chronic mTBI [14, 15] studies confirmed that the relative disturbances of regional CBF were successfully detected by ASL. Furthermore, Ge et al. attempted to evaluate regional CBF changes after chronic mTBI by a resting-state ASL and found significant reductions in mean bilateral thalamic CBF, which was significantly correlated with the simultaneous neuropsychological tests, including measures of learning and memory, response and processing speed, and executive function and verbal fluency [16]. However, the CBF changes in multiple brain regions and the association with neuropsychological cognitive results differ largely across studies. In addition to the limited sample size and the large differences among the subjects, an important technical reason may be the influence of partial volume effect (PVE) [17]. Although PVE was considered in a few PET, SPECT, or ASL studies in mTBI, most studies did not include correction for gray matter volume (GMV), leading to biased results of decreased glucose metabolism and CBF in mTBI patients.

As a reflection of neuronal activity, the CBF in different regions of the brain is not independent. Instead, the CBF in brain regions from the same functional network may change synchronously to achieve the network's function [18]. Melie-Garcia et al. showed that the highest synchronous fluctuations of CBF occur between homologous cortical regions, and functional networks constructed by CBF connectivity have similar network characteristics as functional networks constructed by anatomical or functional connectivity [19]. The alterations of ASL-CBF connectivity have been explored in healthy subjects [20] and diverse diseases, such as schizophrenia [21] and autism spectrum disorders [22]. However, to our knowledge, no studies have evaluated the application of ASL-CBF connectivity in mTBI, and the ASL-CBF connectivity alterations in mTBI patients remain unknown.

In the current study, we utilized ASL-MRI to clarify the pattern of CBF changes and the associations between CBF changes and cognitive impairment in acute mTBI patients. Furthermore, we investigated whether the brain region with abnormal CBF also exhibited CBF connectivity changes in acute mTBI patients. Specifically, to exclude the effect of cortical atrophy on the CBF results, we used information from coregistered structural MRI data to correct ASL-MRI data as well as for GMV variations. We hypothesized that the acute mTBI patients would have different cerebral perfusion and CBF connectivity compared with healthy control (HC), and these changes may be associated with cognitive impairment after mTBI.

## 2. Materials and Methods

### 2.1. Participants and Procedure

The investigation was approved by the Ethics Committee of Nanjing Medical University. All individuals provided informed written consent for their clinical data to be used for the research purposes in the study.

A total of 50 patients with mTBI (recruited from the Emergency Department of Nanjing First Hospital) and 44 HC subjects were included in our study. The inclusion criteria were as follows: (1) age (18-60 years) and right-handedness; (2) a history of a closed head injury with posttraumatic amnesia of less than 24-hour duration, no more than 30 minutes of loss of consciousness, or recorded changes of mental status (i.e., dazed, confused, and disoriented); and (3) an initial Glasgow Coma Score (GCS) of 13-15 obtained by the emergency or ambulatory care staff after injury. The exclusion criteria were as follows: (1) a poor quality of the imaging data, (2) MRI contraindications, (3) neuropsychological disease before injury, (4) history of drug or alcohol abuse, and (5) prior brain injury or other neurologic disease (i.e., epilepsy, stroke, and somatic disorders) that would affect the study results. Specially, all mTBI patients were required to undergo CT scans as a part of their clinical evaluation. Based on these criteria, five patients and two healthy subjects were excluded. Thus, 45 patients with clinically defined mTBI (23 males and 22 females, age range: 20-60 years) were included in this study, with a mean interval of 2.79 days (range: 0-7 days) between trauma and MR imaging findings. All of 42 HC subjects (19 males and 23 females, age range: 20-59 years) were finally enrolled, and they were confirmed to have no brain diseases according to patient history and imaging. The two groups had no significant demographic differences in age, sex, or education level. The clinical neurocognitive state of all participants of psychosis was quantified with the Montreal Cognitive Assessment (MoCA) [23] that evaluated several aspects of cognitive function, including visuospatial/execution, attention, naming, language, abstraction, memory (short-term immediate and deferred recall), and localization [24]. All subjects underwent the same MRI scan and cognitive function assessment within seven days postinjury. The demographic characteristics and outcome clinical measurements of mTBI and HC subject groups are shown in Table 1.

### 2.2. MRI Data Acquisition

MRI was performed using a 3.0-T MR system (Ingenia, Philips Medical Systems, Netherlands) and an eight-channel digital head coil receiver. During the MRI scans, earplugs were used to reduce scanner noise, the tight but comfortable foam padding was used to minimize head motion, and participants were instructed to close their eyes and rest peacefully. The resting-state perfusion imaging was performed using a 2D-pseudocontinuous arterial spin labeling (pCASL) sequence (repetition time = 4000 ms; echo time = 11 ms; label duration = 1650 ms; postlabel delay = 1600 ms; flip angle = 90°; field of view = 240 mm × 240 mm; slice thickness = 5 mm with 10% gap; matrix = 64 × 64; 20 axial slices; total scan duration = 4 min and 08 s). Finally, each subject contained 60 volumes used as 30 label-control image pairs. Sagittal 3D T1-weighted images were acquired using a three-dimensional turbo fast echo (3D-TFE) T1WI sequence (repetition time = 8.1 ms; echo time = 3.7 ms; flip angle = 8°; field of view = 256 mm × 256 mm; acquisition matrix = 256 × 256; slice thickness = 1 mm; gap = 0 mm; 172 sagittal slices; total scan duration = 5 min and 28 s).

### 2.3. MRI Data Processing and CBF Calculation

The pCASL data was processed to generate CBF maps using ASL data processing toolbox: ASLtbx (https://cfn.upenn.edu/~zewan) [25]. The detailed CBF maps calculation procedures have been described in the previous study [26, 27]. Statistical parameter mapping software (SPM8) (http://www.fil.ion.ucl.ac.uk/spm/software/spm8/) and ASL data processing toolbox were used to analyze the ASL image data. Control and label ASL images were rearranged and adjusted to correct head movement. The SPM8 software was used to perform a nonlinear transformation on the CBF images of 42 HC subjects, which were coregistered with the PET-perfusion template in the Montreal Neurological Institute (MNI) space. The MNI-standard CBF template was defined as the average coregistered CBF images of 42 HC subjects. The CBF images of all participants, including mTBI patients and HC subjects, were then coregistered to the MNI-standard CBF template. Each coregistered CBF was removed from the nonbrain tissue and spatially smoothed with a GAUSSIAN of 8 mm × 8 mm × 8 mm FWHM. Normalization was performed by dividing the cerebral blood flow per voxel by the average cerebral blood flow across the entire brain [28].

### 2.4. GMV Calculation

The GMV of each voxel was calculated using SPM8. The structural MR images were segmented into white matter (WM), gray matter (GM), and cerebrospinal fluid (CSF) using the standard uniform segmentation model. The segmented images were used to create a custom DARTEL template, which was then normalized to the Montreal Neurological Institute (MNI) space. Then, the exponential lie algebra (DARTEL) technique was used to perform nonlinear deformation of GM concentration images and resampling them to the voxel size of 1.5 mm × 1.5 mm × 1.5 mm [29]. The GMV of each voxel was obtained by multiplying the GM concentration graph by the nonlinear determinant obtained by the spatial normalization step. Then, the GMV images were smoothed with a Gaussian kernel of 6 mm × 6 mm × 6 mm FWHM. Finally, after spatial preprocessing of the data, the normalized, modulated, and smooth GMV maps were used for statistical analysis.

### 2.5. CBF Connectivity Analyses

Referring to the previous research methods on CBF connectivity [19], in order to detect whether CBF connectivity abnormalities also exist in brain regions with CBF changes in mTBI patients, the clusters with significant group differences in the CBF were selected as seed (regions of interest) ROIs. The CBF values for each ROI within each subject were extracted from a separate CBF map. For each group, a multiple regression model was used to calculate the CBF connections between each ROI seed and all other voxels in the entire brain between individuals with gender and age as confounding covariates. These statistical analyses were used to identify voxels in each group with a positive or negative correlation between CBF values and CBF values for each seed ROI. Multiple comparisons were corrected using FDR correction (*p* < 0.01). For each ROI, the CBF-connected graphs of the two groups were merged into a spatial mask, and the CBF of each voxel was then related to the CBF of the two sets of ROI. For any pair of voxels, the CBF correlation between the two groups may have different slopes, reflecting the difference in CBF connectivity between the two groups. To map voxels that show significantly different CBF correlations between the acute mTBI patients and HC subjects for each seed ROI, a specific T comparison was established within the spatial mask of the CBF connectivity map of ROI. Here, the specific *t*-comparison refers to a two-sample *t*-test analysis of the differences in CBF connectivity between each ROI and all other voxels in the brain in the combined spatial mask between mTBI and the control group.

### 2.6. Correlations Analysis

To investigate the relationships between clinical cognitive parameters of mTBI patients and regional CBF values and CBF connectivity, the ROI-based correlation analysis with cognitive assessment scores was performed for the mTBI patient group using Pearson's correlation analysis, corrected for age, sex, and educational level. The significance threshold of correlation analysis was set at *p* < 0.05.

### 2.7. Statistical Analysis

Two-sample *t*-tests and *χ*^2^ tests were used to analyze the differences in the demographic and clinical data between the mTBI patients and healthy controls by the SPSS 19.0 software package (*p* < 0.05 was considered to be significant). For the normalized CBF and CBF connectivity analyses, a group comparison between the HC group and mTBI group was performed using a two-sample *t*-test, corrected for age, sex, and education level. The significance statistical threshold was set as *p* < 0.01, using false discovery rate (FDR) correct. For each subject, normalized CBF of each cluster with significant differences between groups was extracted for region of interest- (ROI-) based analysis. In order to exclude the influence of GMV on the CBF comparison, we repeated the voxel-based CBF analysis by taking the GMV of each voxel as an uninterested covariable.

## 3. Results

### 3.1. Participants and Clinical Data

Demographics and clinical cognitive assessment of the subjects are summarized in Table 1. Differences in age, sex, and education level between groups were not significant (all *p* > 0.05). In this study, the major mechanism of trauma was motor vehicle collision injury (traffic accidents) [20 of 45 patients (44.4%)], followed by fall from variable heights which was the last [17 of 45 patients (37.8%)] and assault [8 of 45 patients (17.8%)]. As expected, acute mTBI patients had significantly lower MoCA scores than HC subjects (*p* < 0.001). Among all subcategories of the MoCA, only the visuospatial/executive (*p* = 0.007), attention (*p* = 0.003), and language (*p* = 0.005) scores in the mTBI group were significantly lower than those in the HC group, but score differences of abstraction, memory, naming, and orientation between mTBI patients and HC group were not significant (*p* > 0.05).

### 3.2. Group Differences in Resting-State Normalized CBF

**Figure 1:**
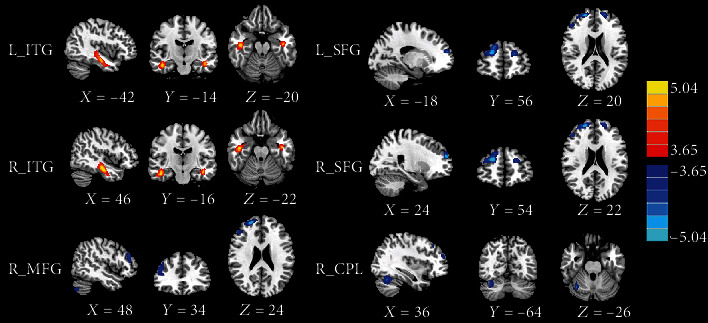
The CBF differences between the mTBI patients and the HC subjects. Compared with HC, the acute mTBI patients showed increased CBF in the bilateral inferior temporal gyrus (ITG) as well as decreased CBF in the bilateral superior frontal gyrus (SFG), right middle frontal gyrus (MFG), and right cerebellum posterior lobe (CPL).

The CBF differences between the mTBI patients and the HC subjects are shown in Figure 1 and Table 2. In voxel-based analysis, the acute mTBI patients showed increased CBF in the bilateral inferior temporal gyrus (ITG) compared with HC. In contrast, these patients also had significantly decreased CBF in the bilateral superior frontal gyrus (SFG), right middle frontal gyrus (MFG), and right cerebellum posterior lobe (CPL).

**Figure 2:**
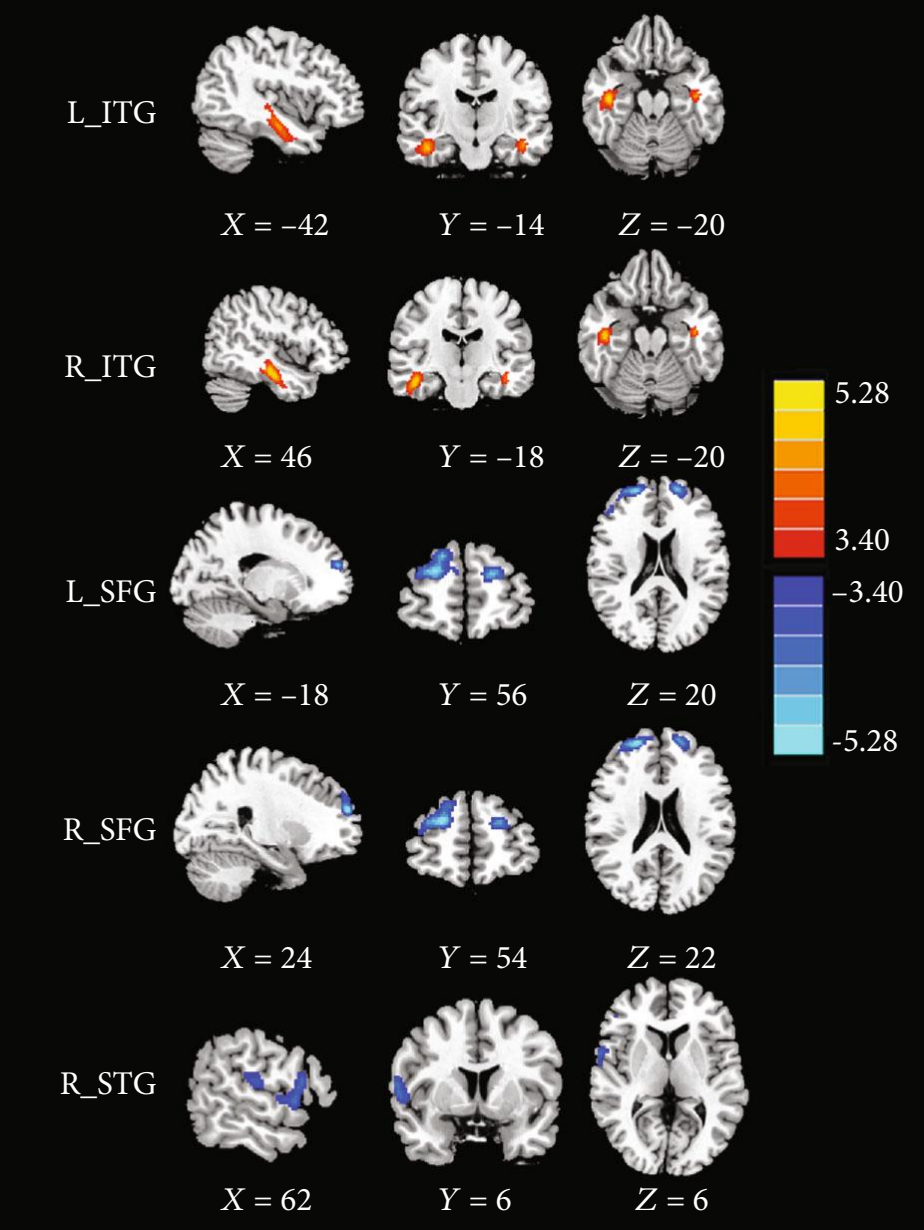
The CBF differences between the mTBI patients and the HC subjects after GMV correction. Compared with HC, the acute mTBI patients showed increased CBF in the bilateral inferior temporal gyrus (ITG) as well as decreased CBF in the bilateral superior frontal gyrus (SFG) and right superior temporal gyrus (STG).

The distribution of the brain regions with significant differences in the CBF after GMV correction is shown in Figure 2 and Table 3. After GMV correction, the acute mTBI patients exhibited significantly increased CBF in the bilateral ITG and decreased CBF in the bilateral SFG (including part of right MFG region), which were the same as the regions without GMV correction. However, the original cluster with reduced CBF in the right CPL disappeared and a new cluster with decreased CBF in the right superior temporal gyrus (STG) were identified after GMV correction.

### 3.3. CBF Connectivity Patterns

**Figure 3:**
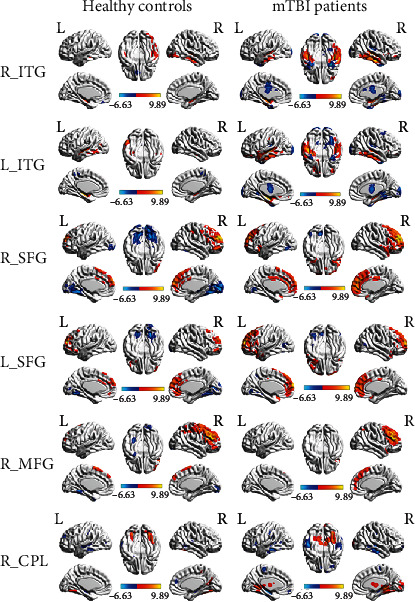
The CBF connectivity maps of six ROIs from the mTBI patients and the HC were displayed, including the bilateral inferior temporal gyrus (ITG), bilateral superior frontal gyrus (SFG), right middle frontal gyrus (MFG), and right cerebellum posterior lobe (CPL).

A total of six ROIs were defined as the seed regions that had significant CBF differences between the mTBI patients and HC subjects. The CBF connectivity maps of each ROI from the two groups are displayed in Figure 3. The left SFG demonstrated similar positive connectivity in both groups but exhibited completely different negative connectivity patterns. The right SFG had CBF connectivity with near regions in the controls, but it exhibited more extensive positive connectivity in mTBI patients. The right CPL showed CBF connectivity with the regions that mainly located in the bilateral cerebral hemisphere in the HC but exhibited more extensive positive and negative connectivity patterns in the mTBI patients. The right MFG had CBF connectivity with near regions in the HC, but it showed more extensive positive connectivity and different negative connectivity patterns. However, the right ITG and the left ITG had a similar connectivity pattern in both groups.

### 3.4. Group Differences in CBF Connectivity

**Figure 4:**
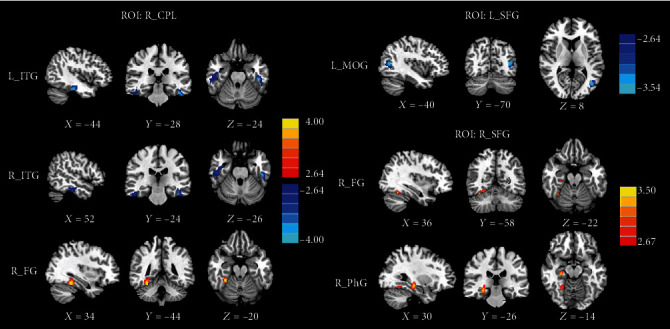
Compared with HC, the acute mTBI patients exhibited decreased negative CBF connectivity between the seed ROI of the right CPL and the right ITG as well as the left ITG and increased positive CBF connectivity with the right FG; the acute mTBI patients showed decreased negative CBF connectivity between the seed ROI of the left SFG and the left MOG; the acute mTBI patients exhibited increased negative CBF connectivity between the seed ROI of the right SFG and the right FG as well as the right PhG.

Group differences in CBF connectivity are shown in Figure 4 and Table 4. Compared with HC, the acute mTBI patients exhibited decreased negative CBF connectivity between the seed ROI of the right CPL and the right ITG as well as the left ITG, but these patients also showed increased positive CBF connectivity between the seed of right CPL and the right FG. Compared with HC subjects, acute mTBI patients also showed decreased negative CBF connectivity between the seed ROI of the left SFG and the left MOG. In addition, acute mTBI patients exhibited increased negative CBF connectivity between the seed ROI of the right SFG and the right FG as well as the right PhG. The seed ROI of the right MFG and the bilateral ITG in CBF did not exhibit any significant differences in the CBF connectivity between both groups.

### 3.5. Correlations between CBF and Neurocognitive Outcome

**Figure 5:**
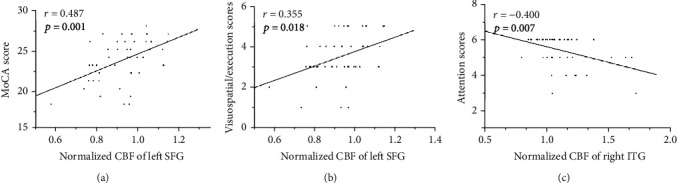
The significant correlations between the CBF changes and the neurocognitive outcome. The MoCA scores (a) and the visuospatial/execution scores (b) were positively correlated with the normalized CBF of the left SFG, respectively (*r* = 0.487, *p* = 0.001; *r* = 0.355, *p* = 0.018). The attention scores (c) were negatively correlated with the normalized CBF of the right ITG in the acute mTBI patients (*r* = −0.400, *p* = 0.007).

The significant correlations between the CBF changes and the neurocognitive outcome (assessed by MoCA) are depicted in Figure 5. Regarding the neurocognitive state, The MoCA scores and the visuospatial/execution scores were positively correlated with the normalized CBF of the left SFG, respectively (*r* = 0.487, *p* = 0.001; *r* = 0.355, *p* = 0.018). In addition, the attention scores were negatively correlated with the normalized CBF of the right ITG in the acute mTBI patients (*r* = −0.400, *p* = 0.007). However, the other regional CBF values and CBF connectivity were not correlated with the cognitive scores.

## 4. Discussion

In the present study, we applied an ASL-MRI technique to investigate the normalized CBF and CBF connectivity changes in acute mTBI patients. To the best of our knowledge, this study is the first to use ASL-MRI to analyze the CBF connectivity patterns of acute mTBI patients. These patients had increased CBF in the bilateral ITG and decreased CBF in the bilateral SFG, right MFG, and CPL. The outcome of the cognitive functional assessment was correlated with the regional normalized CBF. The CBF of the left SFG and right ITG was correlated with the cognitive scores, respectively. More importantly, the CBF connectivity of the bilateral ITG and FG, right PhG, and left MOG was also impaired in acute mTBI patients.

We identified decreased CBF in the bilateral SFG and right MFG, the key regions of the prefrontal lobe, consisting in prior studies in mTBI using ASL-MRI, PET, or SPECT [14, 30, 31]. The frontal lobes are highly evolved and regulate complex behaviors in healthy subjects, which is involved in complex cognitive functions such as planning complex cognitive behaviors, personality expression, decision-making, and regulating social behavior, so hypofrontality may contribute to the clinical symptoms in mTBI [32]. Meanwhile, our result showed that the CBF of the left SFG was positively correlated with the MoCA scores and the visuospatial/execution scores, suggesting that the hypoperfusion in the left CBF may contribute to cognitive deficits in the acute mTBI. In the present study, we also identified decreased CBF in the right CPL, which is involved in the cerebellum network. Although the cerebellum is primarily involved in the function of the motor actions and control, recent studies suggested that the cerebellum is also involved in multiple functions, including cognitive and affective processing, motor-related processing, the experience of thirst, and pain-related processes [33]. Using SPECT, Micarelli et al. have demonstrated that mTBI patients at the acute stage had abnormal CBF in specific regions of the cerebellum, and altered cerebellar CBF was linked to cognitive function [34], confirming the involvement of cerebellum in mTBI. In contrast with the above results, we did not identify any significant correlation between the CBF values in the region of cerebellum and the cognitive scores. The possible reason for this heterogeneous result may result from the differences among the subjects and the experimental methods. Nevertheless, the association of the cerebellum with cognitive impairment after mTBI has not been substantially elucidated and needs to be corroborated in future studies.

In line with previous findings [8, 15], we also identified increased CBF in the bilateral ITG in acute mTBI patients. The ITG is involved in the processes of multimodal sensory integration and visual perception and has been demonstrated volumetric atrophy in mTBI [35]. One possible explanation for the increased CBF in the temporal lobe may result from a short-term compensatory mechanism during the first few days' postinjury, although it has been postulated that the trauma of brain alters cerebrovascular reactivity within the brain. Specially, the increased CBF in the right ITG was found to be negatively correlated with the attention scores in acute mTBI patients, which may explain the deficits of some cognitive function in mTBI. Contrarily, Newberg et al. identified significantly lower CBF levels in the right temporal lobes of mTBI patients using SPECT [36]. In addition, one study found that children with mTBI with persistent posttraumatic symptoms had more severe medial temporal lobe hypoperfusion than those without persistent posttraumatic symptoms [37]. All these studies suggested that the altered CBF in the ITG may underlie the deficits in some cognitive function or posttraumatic symptoms in mTBI. Nevertheless, the specific clinical implication for the altered CBF in the ITG is unclear and needs to be further investigated.

In order to eliminate the effect of GMV, we repeated the voxel-based CBF analyses corrected for the GMV of each voxel. After GMV correction, the mTBI patients at the acute stage exhibited significantly increased CBF in the bilateral ITG and decreased CBF in the bilateral SFG, which were the same as the regions without GMV correction. These findings indicate that the changes of CBF in these regions may be independent of changes in GMV. However, the original cluster with reduced CBF in the right CPL disappeared and a new cluster with decreased CBF in the STG were identified after GMV correction, suggesting that the changes of CBF in the two regions may be related to GM atrophy, either secondary to GM atrophy or compensated for by GM atrophy.

Connectivity alterations in mTBI patients have been extensively studied by MRI techniques, such as the anatomical connectivity derived from diffusion tensor MRI, the structural connectivity derived from structural MRI, and the functional connectivity derived from functional MRI. However, to our knowledge, no study has investigated the CBF connectivity in mTBI. Despite both blood-oxygen level-dependent (BOLD) connectivity and CBF connectivity measure functional correlations between brain regions, they are calculated by different methods and also represent different physiological meanings. The BOLD connectivity was obtained by measuring the temporal correlation of BOLD signal fluctuations between individual brain regions. However, the CBF connectivity was obtained by calculating the correlation coefficient of CBF between brain regions of a group of individuals [38]. For a group of individuals, we can get multiple BOLD connectivity values (a value for an individual), but only one CBF connectivity value could be obtained. The BOLD connectivity represents the synchronization of neural activity between brain regions, while CBF connectivity reflects the coordination of perfusion or metabolism between brain regions. It is worth pointing out that, compared with BOLD connectivity affected by cerebral blood volume, cerebral oxygen metabolism rate, and other physiological parameters, CBF connectivity is only regulated by regional CBF, which has a clearer physiological implication [19]. In the current study, we identified CBF disconnections between the left SFG and the left MOG and between the seed ROI of the right SFG and the right FG as well as the right PhG. The MOG and FG are visual network brain regions, and the PhG is an important structure of hippocampal function, whose damage can cause abnormal emotional and cognitive behaviors [39]. Thus, the disconnection between bilateral SFG and these regions may be linked with the functional deficits in cognitive integration in the acute mTBI patients. Some recent resting-state fMRI BOLD connectivity studies has identified disconnection functional coupling between the prefrontal cortex and these regions [40, 41], which are consistent with our results. In addition, the CBF disconnection between the right CPL and bilateral ITG as well as the right FG was also been identified in mTBI patients, suggesting a disruption of the cerebellar-subcortical-cortical loop, which is partly consistent with the previous resting-state functional connectivity studies that indicate the CPL-FG disconnection and the abnormality of the cerebellum network in mTBI [42–44]. The disconnection of the cerebellum may be related to the functional deficits in cognitive integration in acute mTBI patients. These findings suggested that the CBF connectivity alterations in CPL may be one important brain characteristic of mTBI at the acute stage.

Several limitations should be acknowledged in the current study. First, due to the strict inclusion and exclusion criteria, we recruited a relatively small sample, which may affect the statistical reliability of our results. We hope to expand the sample size and obtain more reliable results in the future studies. Second, all the acute mTBI patients enrolled in this study suffered from complicated injury mechanisms that will influence our interpretation. Subgroup analysis based on different injury mechanisms may help to more accurately describe the pathogenesis of cognitive impairment in patients with mTBI. Furthermore, the CBF connectivity was calculated by analyzing interregional CBF correlations between subject groups (with only one CBF connectivity value for each group) and cannot be used to compare connectivity differences between any two subjects because CBF connectivity is a group-level measure, not an individual-level measure. This measure cannot be used to analyze correlations with clinical or cognitive parameters. The development of ASL techniques with higher temporal resolution and the calculation of CBF connectivity may help solve this problem. Finally, only the brain regions with significant differences in CBF between groups were selected as ROIs for CBF connectivity analysis, which may lead to the omission of CBF connectivity changes between brain regions with normal CBF values. A data-driven approach to whole-brain CBF connectivity analysis will be considered in our future study.

In summary, this study identified the CBF changes in multiple cortical and subcortical regions using ASL-MRI, which may underlie deficits in cognitive function of mTBI patients at the acute stage. Disconnection of CBF connectivity of the bilateral SFG and the right CPL in acute mTBI was investigated for the first time. In our study, both the changes in regional CBF and the disorder of CBF connectivity highlight the need to study the underlying neuropathology of cognitive impairment of the acute mTBI patients from the perspective of the regional and interregional characteristics of the resting-stage CBF.

## Figures and Tables

**Table 1:** Demographic and clinical characteristics of the mTBI patients and healthy controls.

Characteristic	mTBI (*n* = 45)	Controls (*n* = 42)	*p* value
Age (years)	41.36 ± 10.74	40.57 ± 9.95	0.725
Education (years)	12.82 ± 2.75	13.00 ± 2.96	0.773
Sex (female/male)	22/23	23/19	0.669
MoCA scores	23.67 ± 2.77	26.21 ± 1.79	<0.001^∗^
Visuospatial/executive	3.49 ± 1.27	4.19 ± 1.06	0.007^∗^
Naming	2.82 ± 0.38	2.86 ± 0.35	0.662
Attention	5.40 ± 0.86	5.86 ± 0.42	0.003^∗^
Language	2.22 ± 0.73	2.62 ± 0.54	0.005^∗^
Abstraction	1.56 ± 0.58	1.71 ± 0.45	0.165
Memory	2.47 ± 1.29	2.98 ± 1.33	0.074
Orientation	5.71 ± 0.45	5.83 ± 0.38	0.180

The data are shown as the mean ± SD. mTBI: mild traumatic brain injury; MoCA: Montreal Cognitive Assessment. ^∗^*p* < 0.05.

**Table 2:** Brain regions with significant group differences in normalized CBF.

Brain regions	BA	Peak MNI coordinates *x*, *y*, *z* (mm)	Peak *T* value	Cluster size (voxels)
mTBI > HC				
L_ITG	20	-42, -14, -20	4.986	472
R_ITG	20	46, -16, -22	5.319	616
mTBI < HC				
R_MFG	46	48, 34, 24	-4.361	332
L_SFG	10	-18, 56, 20	-4. 499	142
R_SFG	10	24, 54, 22	-5.299	404
R_CPL	—	36, -64, -26	-4.514	276

Thresholds were set at a corrected *p* < 0.01 corrected by FDR criterion. MNI: Montreal Neurological Institute; mTBI: mild traumatic brain injury; HC: healthy control; ITG: inferior temporal gyrus; SFG: superior frontal gyrus; MFG: middle frontal gyrus; CPL: cerebellum posterior lobe.

**Table 3:** Brain regions with significant group differences in normalized CBF with GMV correction.

Brain regions	BA	Peak MNI coordinates *x*, *y*, *z* (mm)	Peak *T* value	Cluster size (voxels)
mTBI > HC				
L_ITG	20	-42, -14, -20	4.717	262
R_ITG	20	46, -18, -20	5.286	545
mTBI < HC				
L_SFG	10	-18, 56, 20	-4.984	231
R_SFG	10	24, 54, 22	-5.588	455
R_STG	44	62, 6, 6	-4.348	163

Thresholds were set at a corrected *p* < 0.01 corrected by FDR criterion. MNI: Montreal Neurological Institute; mTBI: mild traumatic brain injury; HC: healthy control; ITG: inferior temporal gyrus; SFG: superior frontal gyrus; STG: superior temporal gyrus.

**Table 4:** Brain regions with significant group differences in CBF connectivity.

ROI	Brain regions	BA	Peak MNI coordinates *x*, *y*, *z* (mm)	Peak *T* value	Voxels
R_CPL	L_ITG	20	-44, -28, -24	-3.941	295
	R_ITG	20	52, -24, -26	-3.296	291
	R_FG	37	34, -44, -20	4.276	157
L_SFG	L_MOG	39	-40, -70, 8	-3.738	172
R_SFG	R_FG	37	36, -58, -22	3.475	112
	R_PhG	36	30, -26, -14	3.571	107

Thresholds were set at a corrected *p* < 0.01 corrected by FDR criterion. CBF: cerebral blood flow; ROI: region of interest; MNI: Montreal Neurological Institute; CPL: cerebellum posterior lobe; ITG: inferior temporal gyrus; FG: fusiform gyrus; SFG: superior frontal gyrus; MOG: middle occipital gyrus; PhG: parahippocampal gyrus.

## Data Availability

The data used to support the findings of this study are available from the corresponding author upon request.
